# Effects of Starter Defect on Energy Release Rate of Three-Point End-Notch Flexure Tested Unidirectional Carbon Fiber Reinforced Polymer Composite

**DOI:** 10.3390/polym12040904

**Published:** 2020-04-14

**Authors:** Fethma M. Nor, Joong Yeon Lim, Mohd Nasir Tamin, Ho Yong Lee, D. Kurniawan

**Affiliations:** 1 Department of Mechanical Engineering, Curtin University, Miri 98009, Malaysia; fethma@curtin.edu.my; 2 Department of Mechanical, Robotics, and Energy Engineering, Dongguk University, Seoul 04620, Korea; hoyong@dongguk.edu; 3 School of Mechanical Engineering, Universiti Teknologi Malaysia, Johor Bahru 81310, Malaysia; nasirtamin@utm.my; 4 Faculty of Engineering, Universiti Teknologi Brunei, Gadong BE1410, Brunei Darussalam; denni.kurniawan@utb.edu.bn

**Keywords:** CFRP, end-notch flexure, delamination, energy release rate, crack profile

## Abstract

The mechanics of damage and fracture process in unidirectional carbon fiber reinforced polymer (CFRP) composites subjected to shear loading (Mode II) were examined using the experimental method of the three-point end-notch flexure (3ENF) test. The CFRP composite consists of [0^o^]_16_ with an insert film in the middle plane for a starter defect. A 3ENF test sample with a span of 50 mm and interface delamination crack length of 12.5 mm was tested to yield the load vs. deformation response. A sudden load drop observed at maximum force value indicates the onset of delamination crack propagation. The results are used to extract the energy release rate, G_IIC_, of the laminates with an insert film starter defect. The effect of the starter defect on the magnitude of G_IIC_ was examined using the CFRP composite sample with a Mode II delamination pre-crack. The higher magnitude of G_IIC_ for the sample with insert film starter defect was attributed to the initial straight geometry of the notch/interface crack and the toughness of the resin at the notch front of the fabricated film insert. The fractured sample was examined using a micro-computerized tomography scanner to establish the shape of the internal delamination crack front. Results revealed that the interface delamination propagated in a non-uniform manner, leaving a curved-shaped crack profile.

## 1. Introduction

Laminated carbon fiber reinforced polymer (CFRP) composites exhibit customizable top-of-the-range properties, making them suitable for structural materials in various engineering applications, including in transportations, infrastructures, energy, marine, and sports and leisure. However, CFRP composites have relatively low interlaminar strength, making them susceptible to delamination damage, that might happen during manufacture or in service [1]. The presence and growth of delamination in CFRP composites may significantly reduce their stiffness, compromising their structural integrity, and even leading to a catastrophic failure. Considering these, delamination resistance of CFRP composites must be measured reliably and be taken into account in the design and manufacture of CFRP composites [1].

The delamination resistance of CFRP composites is commonly represented by interlaminar fracture toughness, which is often identified as critical strain energy release rate (G_C_). In determining G_C_, energy based linear elastic fracture mechanics are the popular application to use [2]. The delamination can happen in an opening mode (Mode I), with the G_C_ for this mode of delamination termed as G_IC_; and the delamination can also happen in a sliding shear mode (Mode II), with the G_C_ is termed as G_IIC_. Studies by experimental, theoretical, and numerical simulations have been done on the expressions for G_IC_ and G_IIC_, with measurement of G_IIC_ said to be the more complicated exercise. For this, a three end-notched flexure (3ENF) test [3] can be applied because it requires simple fixtures. Moreover, it is a standard test for the purpose, according to Japan Industrial Standards [4].

It was reported that the G_IIC_ from 3ENF test was affected by the thickness of the specimen. This situation happens when the load used is either at the first non-linear point of the load vs. displacement curve (where crack initiates) or at the maximum load of the load vs. displacement curve [5,6,7]. The effect of thickness on G_IIC_ was suggested to be due to friction between surfaces of the starting defect [5] or due to fiber bridging in the generated pre-crack in Mode II [7].

G_IIC_ from 3ENF test was also reportedly affected by the thickness of the insert film used to make the starter defect of the 3ENF specimens. Tanaka et al. [6] reported that, when the thickness of the insert film varied from 7.5 to 25 μm, the obtained G_IIC_ values significantly differed. Hence, Japan Industrial Standards recommends using an insert film of less than 30 μm thickness between the two middle laminae during lay-up process in the manufacture of the CFRP composite.

Generally, G_IIC_ is influenced by the bond strength at the fiber/matrix interface, according to experimental evidence [8,9,10,11,12]. This means G_IIC_ represents the critical strain-energy release rate for crack growth from the insert film. However, analysis of this situation is still needed. This is because of the effect of the bond strength at the fiber/matrix interface on the stress distribution within the resin-bags in front of the insert film, where the crack initiation and growth is not well understood. To further understand this, this study examines the physical aspect of crack initiation and growth of a unidirectional CFRP composite experiencing delamination under Mode II by 3ENF test. Two types of starter defects were tested and analysed for this purpose, i.e., insert film in the middle laminae and pre-cracking by Mode II load.

## 2. Materials and Methods 

The CFRP composite was a [0^o^]_16_ laminate from manually laid up unidirectional laminae of carbon/epoxy prepreg (Structil). Two types of samples having different starter defects were investigated. The first one had insert film put in the middle plane between two laminae during lay up as the starter defect, termed as CFRP1. The PTFE insert film was 23 µm in thickness in order to comply with the European Structural Integrity Society (ESIS) TC4 protocol for Mode II interlaminar fracture toughness of unidirectional fiber-reinforced polymer composites [3]. The second one is pre-cracked samples loaded by Mode II test to grow the delamination beyond the thin film starter defect tip, termed as CFRP2.

**Figure 1:**
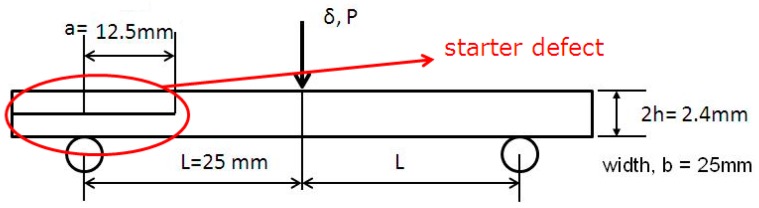
End-notched flexure test set up with sample having starter defect.

The three-point end-notch flexure test was carried out according to the ASTM D790 and ESIS 3ENF test protocols. The 3ENF test sample was 25 mm wide, 70 mm total length, and the initial crack length was 12.5 mm. The ratio of the length of the starter defect to the half span was fixed at 0.5. The 3ENF tests were carried on an Instron 8501 universal testing machine using a 5 kN load-cell, with 1 mm/min crosshead displacement, and with load applied at the mid-point (Figure 1).

For every 3ENF test, the load vs. displacement (P–δ) data were recorded. The Mode II interlaminar fracture toughness was calculated from the initial crack length and the load vs. displacement curve using the highest load and deflection level as in Equation (1).

(1) $$G_{IIc}=\frac{9a^{2}P_{c}^{2}}{16Eb^{2}h^{3}},$$

where *a* is the initial crack length, *P_c_* is the critical load, *h* is half of the overall thickness, *b* is the width of the specimen, and *E* is stiffness (Equation (2)).

(2) $$E=\frac{\left(2L^{3}+3a^{3}\right)}{8Cbh^{3}},$$

where *L* is half the distance between supports, *a* is the initial crack length, *b* is the width of the specimen, *h* is half of the overall thickness, and *C* is the compliance.

The 3ENF test was done twice for each sample, with the G_IIc_ values determined from the point at which the load vs. displacement curve deviates from linearity, G_IIc-NL_, and also at maximum load, G_IIc-max_.

Samples then were prepared for the material characterizations. The materials characterizations were focused on the type of the internal crack profile using a micro-computerized tomography (CT-scan) and confirmed using a scanning electron microscope (SEM). The CT-scanner used to examine the composite was a SkyScan 1076, a high resolution, low dose microtomograph for in-vivo 3D-reconstruction, with spatial resolution of up to 15 µm. The samples were placed on a 68 mm wide sample holder. The resolution was set at 35 mm, an averaging of three was employed together with a filter of 0.5 mm aluminum, a rotation step of 0.8, and the rotation angle was 360^o^. The scans in this study were conducted with x-rays at a voltage of 90 kV. Approximately 500 scan slices were taken for each sample. The three-dimensional reconstruction was done using NRecon and the visualization and analysis was done using CTan, CTVol, and CTVox, as provided by SkyScan.

## 3. Results and Discussion

### 3.1. Mode II Strain Energy Release Rate of the Unidirectional CFRP Composite

**Figure 2:**
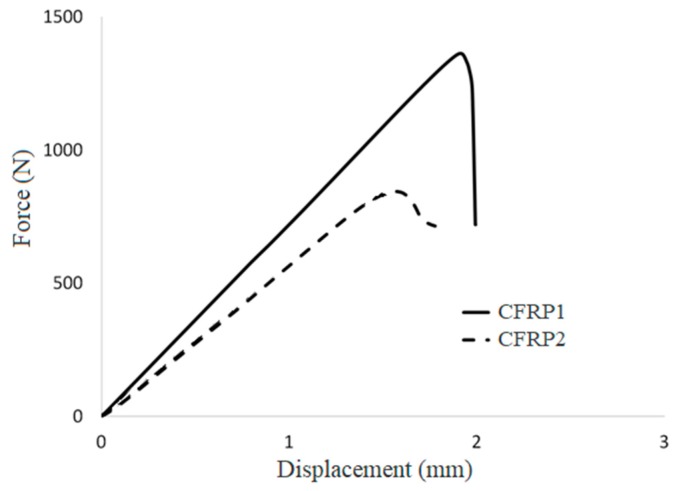
Typical load vs. displacement curves of the carbon fiber reinforced polymer (CFRP) composites under three-point end-notch flexure (3ENF) test.

Typical load vs. displacement curves resulted from the 3ENF test on the particular unidirectional CFRP composites with two types of starter defects are depicted in Figure 2. The CFRP composite with insert film as starter defect (CFRP1) showed that force increases linearly, reaches a peak value, and experiences a sudden load drop. This brittle failure response, in which sudden drop occurred after the composite reached its fracture point, is typical for CFRP composites under flexural loading [13,14,15]. CFRP composites may at times exhibit stiffness degradation (decreasing slope of the curve) just before failure [15]. However, this particular composite seemed to not show such a phenomenon. For CFRP composite with mode II pre-crack as starter defect (CFRP2), the force linearly increases, followed by a nonlinear increase to reach the peak value, and declines nonlinearly within short displacement range after reaching peak value, then finally drops. The CFRP2 samples seem to exhibit degradation of stiffness just before failure [15].

As mentioned above, some CFRP composites exhibit similar stiffness degradation just before failure. Previous observation on unidirectional CFRP composite under flexural loading found that internal failure actually occurs—it even begins at load of about 60% of the failure load, even for composites which show brittle fracture where no detectable nonlinearity was present [13,14]. The researchers proved it by measuring acoustic emission signal transmitted by the flexure loaded specimen, and at the same time captured its images in situ. The stages of failure mechanism of flexural loaded unidirectional CFRP was perceived to be initiated by fiber breakage (at about 60% of failure load), followed by matrix cracking alongside broken fibers, followed by partial delamination (just before failure load), and finally abrupt crack propagation (at and beyond failure load) [13]. Thus, failure mechanism of CFRP1 which showed brittle failure response might also initiate from fiber breakage, like what seemingly happened to CFRP2. Although macroscopic observation of load-displacement measurement did not detect the initial stage of failure. Whether this initial analysis is correct still needs to be proven with more data. It should be noted, however, that there is no report on similar failure mechanism analysis on unidirectional CFRP composites under end-notch flexure test. Nor is there a report that analyses why certain CFRP composites show stiffness degradation, while others do not.

**Figure 3:**
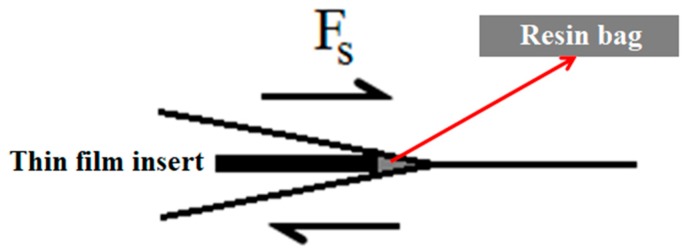
Resin bag supposed to feature in sample with thin film insert as starter defect.

In terms of point of failure, CFRP1 failed at a higher peak load and at a higher elongation at break than CFRP2. The former’s slope was also higher than that of the latter. There are some possible reasons for CFRP1 (sample with insert film as starter defect), showing both higher stiffness and strength compared to its Mode II pre-cracked counterparts (CFRP2). First, there are resin bags adjacent to the thin film in CFRP1 (Figure 3), while there is none in CFRP2. This resin bag is vested in CFRP1, considering the fabrication method, in which the thin film used to make the end-notch region was actually slotted within stacks of pre-pregs [16,17]. The consequence of these resin bags is the occurrence of sites with lower fiber fraction ratio (higher matrix content). Hence, the higher elongation at break shown by CFRP1. The second reason for the higher stiffness, strength, and elongation at break of CFRP1, compared to those of CFRP2, is that the interface adhesion between the matrix and carbon fiber in the former was higher than it was in the latter. The cause for this difference in interfacial adhesion might be in part from the subpar performance of the CFRP2. If the first reason is true, in which there are sites with lower fiber volume fractions in CFRP1, then CFRP2 which contains no such site should exhibit higher stress and strain. Causes for this include the induced microcracks at the fiber-matrix interface and more areas of shear stress concentration, both of which may possibly occur if the crack front is wider than when the starter crack was a thin film. In other words, the crack front is curved or nonlinear, as opposed to its linear initial condition, similar to the crack front feature in previous reports [16,18].

By using Equation (1), the fracture toughness or critical energy release rate under mode II fracture (G_IIC_) was calculated. It was calculated that the G_IIC_ for CFRP1 was 1.98, while the G_IIC_ for CFRP2 was 0.96. Peak load value was used to determine the corresponding critical load, considering CFRP2 showed small region of nonlinearity while CFRP1 did not. The initial G_IIC_ for the sample with insert film as starter defect (CFRP1) was higher than that for the sample with Mode II precrack as starter defect (CFRP2). Referring back to Equation (1), considering crack length (*a*) and sample size (*b* and *h*) were constant, the difference in G_IIC_ values was due to the Young’s modulus (*E*) and the critical load (*P_C_*) of the CFRP composites. CFRP1 exhibited slightly higher Young’s modulus and much higher critical load and, hence, its higher G_IIC_. This result is in agreement with previous studies [3,6,16].

Presence of resin bags in CFRP1, as mentioned above, might be the cause [17]. Resin bag’s fiber volume fraction is low, which means it has a higher matrix content. The higher G_IIC_ of CFRP1 might be reflected from this corresponding higher matrix content at the resin rich crack tip. Additionally, crack front condition of the CFRP2 might be influential as well. As mentioned above, there is possibility that the crack front of CFRP2 might be curved, which means the crack front is longer than the initially linear crack tip. Damage zone developed at the crack tip dissipates some portion of the total external energy the sample was subjected to (by shear loading), before macroscopic propagation [16]. The longer the crack tip, as in CFRP2’s supposedly curved crack front, the more strain energy dissipated prematurely and did not reflect in the bulk composite’s force-displacement curve. Hence, the lower G_IIC_ of CFRP2.

Another trial was made on the CFRP2 sample by subjecting it to another round of 3ENF test, herein termed CFRP2’ for convenience. The load vs. displacement curve of CFRP2’ is similar to that of CFRP2, including the nonlinear decrease after the peak point (i.e., stiffness degradation). In terms of critical energy release rate under Mode II fracture, the G_IIC_ of CFRP2’ was similar to CFRP2. A possible explanation for this similar Young’s modulus, strength, elongation at break, and G_IIC_ is the crack front of CFRP2’ resembled that of CFRP2, which is curved. This similarity was a preferred indication of reproducibility, when mode II pre-crack was used as starter defect for 3ENF test of unidirectional CFRP composite.

Regarding the G_IIC_ values obtained by the 3ENF test, this study found that unidirectional CFRP composite with mode II pre-crack as starter defect showed a more conservative G_IIC_ value, compared to the value shown by composite with insert film as starter defect. Previous works on 3ENF tests of unidirectional composites [3,6,16], which also recorded similar findings, stated that the obtained G_IIC_ value was not sensitive to thin film thickness (prior to making the pre-crack) and length of the pre-crack. This current study adds that the G_IIC_ value is also insensitive to the number of pre-cracks that the sample was preconditioned by.

Among factors which were said to be influential to the G_IIC_ is the thickness of the thin film as starter crack. It was recommended that the thin film insert’s size should be less than 30 µm [4] in order to minimize its effect on G_IIC_ (due to the occurrence of resin bags). The thickness of the insert film used to make the unidirectional CFRP composites in this study was 23 µm, so it complied. Still, the discrepancy between the G_IIC_ values of CFRP1 and CFRP2 was apparent. So, it was more on the less performing CFRP2, perceived here due to development of damage zone in the form of microcracks at curved crack front, which were longer than if it was linear.

Another factor that influenced G_IIC_ was the selection of critical load; be it the one where nonlinearity starts, or the one at peak point [16]. When the former was selected as the critical load, it resulted in even lower G_IIC_ for CFRP2. This study used the peak load as the critical load, considering that at the peak load, the composite can still withstand the external load it is subjected to, even though the damage zone already initiates. Friction is another factor which might affect the G_IIC_. This friction occurs because shearing happens, and the end-notch surfaces are in contact one another. Yet, a report showed that friction does not influence Mode II fracture test [18]. This means, friction also did not affect the results in this study. Overall, taking into account the conservativeness and consistency of the G_IIC_ results, this study recommends the use of pre-crack as a starter defect in 3ENF test of unidirectional CFRP composites whenever applicable, especially for design purposes.

### 3.2. Crack Front Profile

In Section 3.1, it was perceived that the crack front profile of CFRP2 was curved. Previous reports actually have stated about this [16,19,20]. However, no evidence was brought up yet, which is understandable considering limitations of currently available imaging technology. X-ray imaging, which is expected to be the enabling technology, still has limitations in which contrast between carbon fiber and epoxy matrix in CFRP is too low, in addition to the relatively low resolution of the system. Thus, detailed information which might be helpful for fracture analysis (e.g., fiber breakage, fiber-matrix interfaces, microcracks, and delamination) is difficult to obtain. This study explores the use of micro CT scan to obtain as much information as possible about the crack front to help analyze the finding related to G_IIC_.

**Figure 4:**
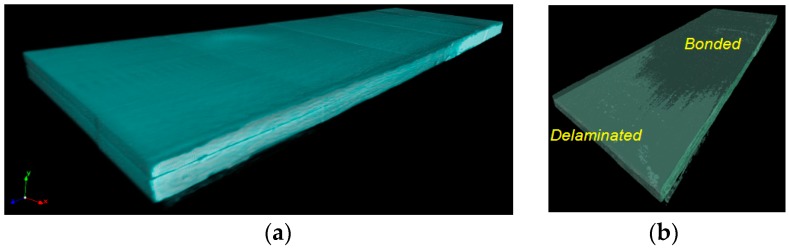
Isometric views of (**a**) the 3ENF tested unidirectional CFRP composite with thin film as starter defect (CFRP1) and (**b**) with its crack front profile visible.

Studies demonstrate that the x-ray microtomography technique using CT scans can be used to reveal matrix damage, such as micro crack and delamination in CFRP composites [21]. Analysis of damage mechanism using this technique is greatly assisted if there is sufficient contrast between matrix and fibers (in terms of x-ray attenuation), so the setting should be adjusted to obtain this. Initial trial was performed on 3ENF tested CFRP composites with insert films as starter defect (CFRP1), and the result was depicted in Figure 4. The starter defect and subsequent damage due to the 3ENF test on CFRP1 could be visualized. The system and setting were capable of getting adequate contrast to differentiate air from the bulk of CFRP1. The rendered image obtained from the micro CT scan is three dimensional and can be treated as one (e.g., sliced to view a certain section). However, the resolution is still too low to clearly differentiate between the fiber from the matrix.

From the available 3D image (one representation is in Figure 4), the cracked CFRP1 features the curved crack front. This micro CT image of curved crack front profile, as the result of 3ENF test on unidirectional CFRP composite, is currently the first one ever reported. A previous report [16] had indirect analysis that crack front of 3ENF tested CFRP was curved, but no image was shown as evidence, and attempts to verify this by sectioning and acoustic emission were unsuccessful.

**Figure 5:**
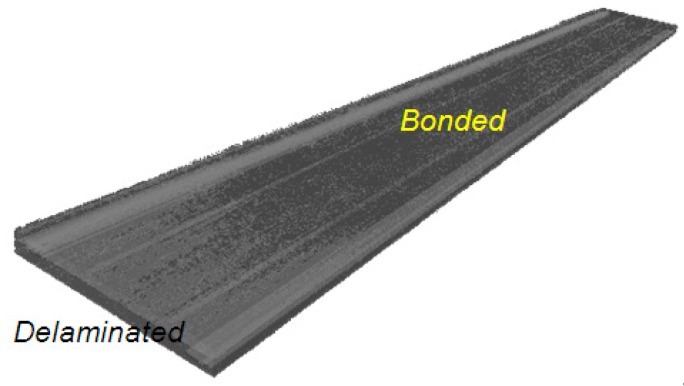
Isometric view of the 3ENF tested unidirectional CFRP composite with Mode II pre-crack as starter defect (CFRP2), with crack front profile shown.

As for 3ENF tested CFRP composites with mode II precrack as starter defect (CFRP2), its crack front also shows a similar curved profile (Figure 5). This finding supports the analysis about the less performing CFRP2, which was perceived due to its curved (which means longer than initially linear) crack front. It should be noted that the shown crack front was obtained after the 3ENF test. The initial crack front is the one obtained after the 3ENF test of CFRP1, thus it corresponds to the curved crack front profile of CFRP1. This means there actually was no apparent change in the length of the crack front for CFRP2, since it started with a curved crack front and ended also with a curved crack front (the length was even slightly reduced). Chances are that the CFRP2 did not show subpar properties, as observed in previous section (judging from load vs. deformation curve alone). It readily showed consistent behavior in terms of G_IIC_. Similar G_IIC_ of CFRP2’ (when CFRP2 was subjected to another round of 3ENF test) from that of CFRP2 verified this. This finding is in line with the current recommendation to use G_IIC_ obtained from 3ENF tests on unidirectional CFRP composites for design consideration for showing conservative and consistent values.

**Figure 6:**
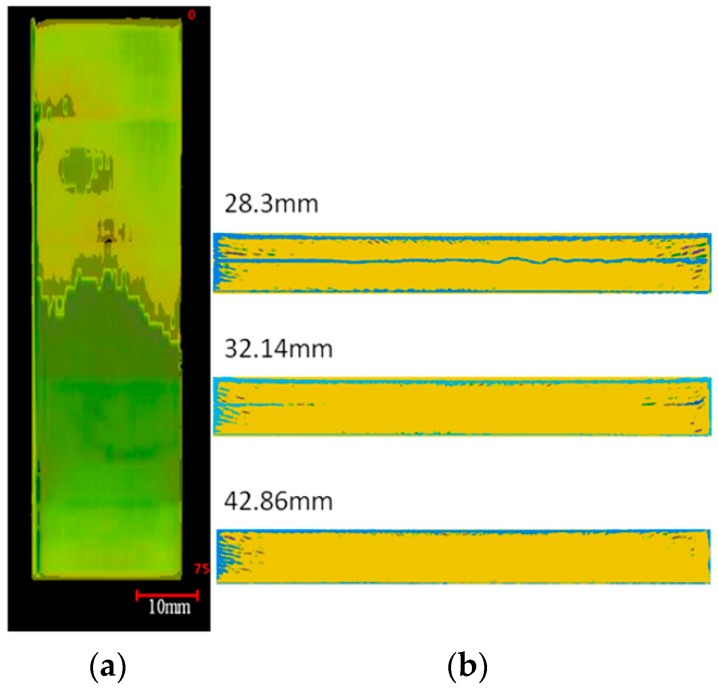
(**a**) Top and (**b**) front views of the 3ENF tested CFRP2 sample [Note: the numbers indicate the distance from the front end].

Clearer view on the damage zone of the CFRP2 sample can be seen from Figure 6 where the top and front views are presented. Wavy crack front when seen from front view is apparent. The insert film used to make the end-notch (starter defect) for the composites was expected to produce flat crack front. However, since the rigidity of thin PTFE film is low, the thin film could not resist the tendency for ply waviness during fabrication [14,15,20]. Seen from the top and front views, it is obvious that the crack front of CFRP2 was curvy, with propagation more towards both edges and less in the center region. The curved crack front was opposite to that observed by Davies et al. [16], who reported that the crack propagation at the specimen center was up to 2 mm longer than on the edges.

**Figure 7:**
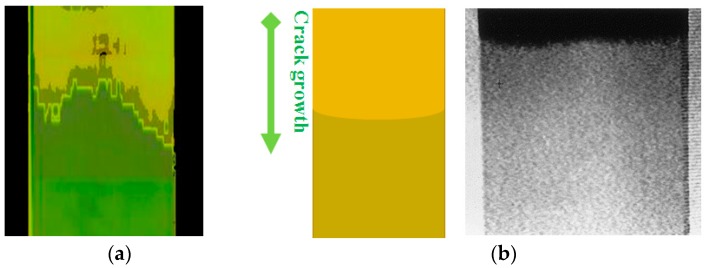
Internal crack profiles of (**a**) Mode II fracture in this study and (**b**) Mode I fracture [19].

Comparing the crack front profile between that obtained from mode II fracture test found in this study and mode I fracture test performed previously [19], the orientation of the curve was the opposite (Figure 7). The propagation pattern that eventually becomes the final crack front profile might be governed by the location of the site of initial crack. Considering the crack front profiles of CFRP1 and CFRP2 reported here, it seems crack initiation started from the edges. The microcracks might initiate from sites with less matrix content (higher fiber volume fraction) at the edges, created when the specimen was sectioned. This observation that the crack initiated at the edges puts a doubt to analysis in Section 3.1, where the cracks initiated due to fiber breakage like what happened to flexure tested CFRP composites [13].

A remark on this 3ENF test, is that since the crack front was curved, and the reading of crack length which commonly is done from the edges might be compromised. The measured crack length might not be representative. The reading can be an overestimation like what was found in this study. In other circumstances, it can also be an underestimated value from samples which feature a crack at the center, propagated more than on the edges. The reading of crack length influences the G_IIC_ value. An example from this study is given in Table 1. The difference in crack length and the corresponding G_IIC_ can be twofold when the reading is taken from the edge, which showed most propagation compared to that when the reading was taken from the center. Thus, when possible, calculation of G_IIC_ which comes from reading of crack length at edges should be avoided, or the deviation should be presented as well. There are ways to obtain G_IIC_ other than from direct reading of the crack length at the edges. One of them is proposed by Tanaka et al. [6], which measures crack shear displacement to calculate G_IIC_. This method was demonstrated to result in non-significant differences in toughness values calculated from equations based on crack length or based on crack shear displacement.

### 3.3. Fractography Analysis

**Figure 8:**
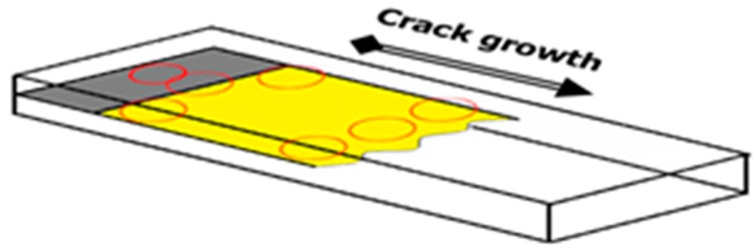
Schematic drawing of the location of the SEM observation on CFRP2 sample.

**Figure 9:**
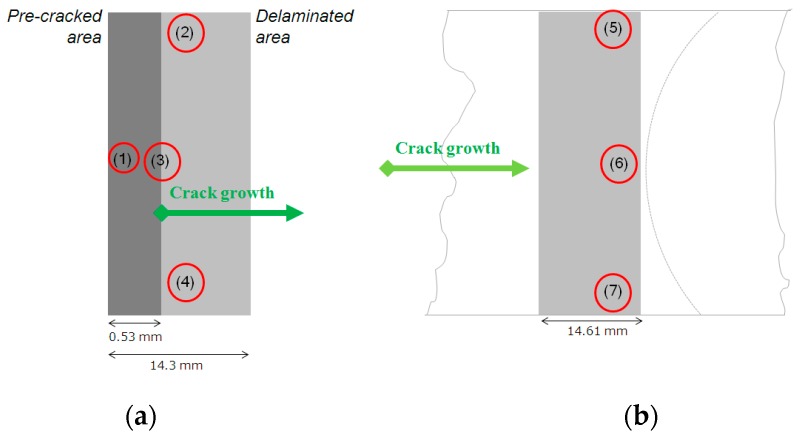
Schematics of top view of the CFRP2 sample showing the locations of SEM observation: (**a**) initial condition and (**b**) after 3ENF test.

Fractography analysis on 3ENF tested CFRP composites was done using scanning electron microscope (SEM). It was intended to complement the analysis on damage mechanisms on the unidirectional CFRP composites. Another intention was to verify the findings from micro CT scans, since the rendered images resolution of the scanned object was not so high. The 3ENF tested samples were opened to reveal the paths of crack propagation. The analysis was done only on the CFRP2 sample, since it featured both the traces of crack front from 3ENF tested CFRP1 and the final crack front of CFRP2 itself. Considering the small area coverage of SEM, the images should be captured from multiple sites of interest within the sample. A schematic drawing showing the locations of the SEM observation taken on the sample is depicted in Figure 8. To help clarify the location, top view of the sample showing the location of SEM observation was also presented (Figure 8). What to observe in Figure 9a is the trace of a crack front left behind by 3ENF tested CFRP1. Four locations were observed for this purpose. Point 1 was at the pre-cracked area (initial) where it included the insert film’s image. Points 2 and 4 show the delaminated area at the edges, while point 3 is at the center. Figure 9b shows the shortest final crack front of the 3ENF tested CFRP2, where points 5 and 7 were at the edges while point 6 was at the center. The longest path of the crack front at edges was not observed due to difficulty in sectioning the sample without forced opening (intended to preserve the crack front).

**Figure 10:**
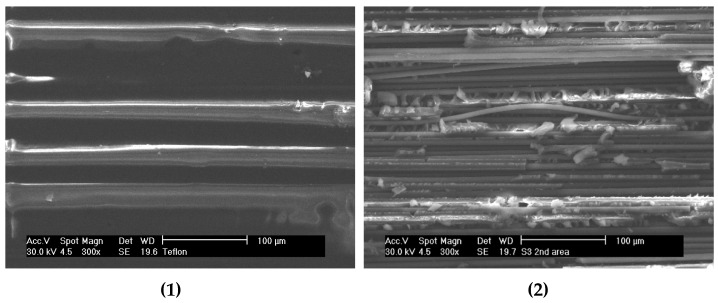

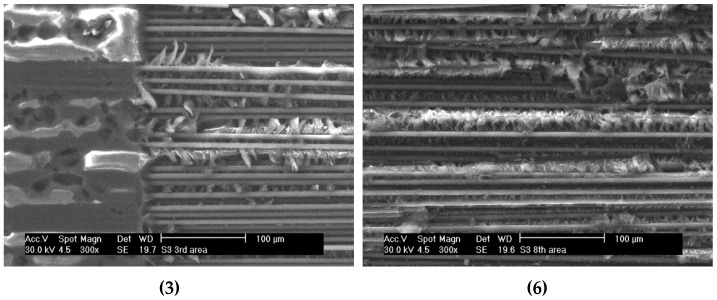
Fractographs of Mode II crack for sample with thin film starter defect. [Note: Number in bracket denotes the location of observation as shown in Figure 9].

Figure 10 shows SEM images of the observed sites within CFRP2. There was no sign of fiber breakage, confirming the analysis that fiber breakage was not the crack initiator. This might indicate that crack initiation started at edges and was initiated by microcracks at the matrix or at matrix-fiber interface. The figures of the damaged zone show that there are hackles formed at the matrix. They are characteristics of Mode II fracture at the matrix [22,23]. There was no sign of shear lip formation, an indicator of ductile matrix [24], thus confirming that the epoxy matrix is of strong and brittle type. Density of hackles is proportional to strain [22]. Figure 10 location 6 showed only slight difference in density of hackles, and a similar trend was found on locations 5 and 7. However, there was the occurrence of pulled fibers at each edge. These evidenced that the applied shear load induced higher strain at the edges than at the center. Indirectly, it can be deduced that the cracks indeed propagated more at the edges than at the center when CFRP1 was 3ENF tested.

At the final crack front of the CFRP2 sample, in particular at the center, it was apparent that hackles at the center were less rotated than those at the edges (Figure 10). This evidenced that the edges were more strained compared to the center. However, it cannot be deduced that for 3ENF tested CFRP2, the cracks propagated more at the center, considering the initial crack front was readily curved.

Overall, the fractographs of the 3ENF tested unidirectional CFRP composite sample supported the finding obtained from the micro CT scan. It was found, albeit through low resolution micro CT images, that the crack front profiles of 3ENF tested CFRP1 and CFRP2 are curved, with more propagation occurred at edges compared to at the center. This fractography analysis complements and confirms the finding from the micro CT scan.

## 4. Conclusions

The initial G_IIC_ of thin unidirectional CFRP composites can be obtained using 3ENF test. The G_IIC_ of CFRP with insert film as starter crack (CFRP1) was higher than that of its Mode II pre-cracked counterpart (CFRP2). It reflects the occurrence of resin bags. Mode II crack propagates nonuniformly throughout the width of sample. Crack at the edges propagates more than at the center. Caution must be taken when including the starter defect for mode II fracture, as taking the G_IIC_ value from samples with thin film as the end-notch may lead to overestimation or underestimation. It was suggested to apply other techniques which are less sensitive to crack length reading errors. Nondestructive evaluation using micro CT scans can be used to examine the crack front profile of unidirectional CFRP composites. With all its limitations, sufficient contrast between constituents of the composites was obtained such that observation on damage zone can be performed. Fractography analysis on the damage zone using SEM confirms and verifies the findings of micro CT scan images.

## Figures and Tables

**Table 1:** G_IIC_ values of samples with crack length readings taken at various positions within the crack front.

Sample Type	Crack Length (mm)	G_IIc_
**CFRP2**		
Minimum	11.3	0.85
Average	12.5	0.98
Maximum	23.5	1.86
**CFRP2’**		
Minimum	11.5	0.81
Average	12.5	0.92
Maximum	19.7	1.57
